# The impact of isocentric shifts on delivery accuracy during the irradiation of small cerebral targets—Quantification and possible corrections

**DOI:** 10.1002/acm2.12854

**Published:** 2020-03-20

**Authors:** Linda J. Wack, Florian Exner, Sonja Wegener, Otto A. Sauer

**Affiliations:** ^1^ Radiation Oncology University of Wuerzburg Wuerzburg Germany

**Keywords:** isocenter, quality assurance, stereotactic radiotherapy, Winston‐Lutz test

## Abstract

**Purpose:**

To assess the impact of isocenter shifts due to linac gantry and table rotation during cranial stereotactic radiosurgery on D_98_, target volume coverage (TVC), conformity (CI), and gradient index (GI).

**Methods:**

Winston‐Lutz (WL) checks were performed on two Elekta Synergy linacs. A stereotactic quality assurance (QA) plan was applied to the ArcCHECK phantom to assess the impact of isocenter shift corrections on Gamma pass rates. These corrections included gantry sag, distance of collimator and couch axes to the gantry axis, and distance between cone‐beam computed tomography (CBCT) isocenter and treatment beam (MV) isocenter. We applied the shifts via script to the treatment plan in Pinnacle 16.2. In a planning study, isocenter and mechanical rotation axis shifts of 0.25 to 2 mm were applied to stereotactic plans of spherical planning target volumes (PTVs) of various volumes. The shifts determined via WL measurements were applied to 16 patient plans with PTV sizes between 0.22 and 10.4 cm^3^.

**Results:**

ArcCHECK measurements of a stereotactic treatment showed significant increases in Gamma pass rate for all three measurements (up to 3.8 percentage points) after correction of measured isocenter deviations. For spherical targets of 1 cm^3^, CI was most severely affected by increasing the distance of the CBCT isocenter (1.22 to 1.62). Gradient index increased with an isocenter‐collimator axis distance of 1.5 mm (3.84 vs 4.62). D_98_ (normalized to reference) dropped to 0.85 (CBCT), 0.92 (table axis), 0.95 (collimator axis), and 0.98 (gantry sag), with similar but smaller changes for larger targets. Applying measured shifts to patient plans lead to relevant drops in D_98_ and TVC (7%) for targets below 2 cm^3^ treated on linac 1.

**Conclusion:**

Mechanical deviations during gantry, collimator, and table rotation may adversely affect the treatment of small stereotactic lesions. Adjustments of beam isocenters in the treatment planning system (TPS) can be used to both quantify their impact and for prospective correction of treatment plans.

## 1 INTRODUCTION

Conventional linear accelerators, unlike dedicated machines such as the Cyberknife, were originally not considered suitable for stereotactic radiosurgery. However, in recent years, high‐precision irradiation of stereotactic lesions using linear accelerators has become wide‐spread, as modern advances in treatment delivery and patient positioning allow for the treatment of increasingly small lesions with the necessary accuracy.^1^, ^2^, ^3^, ^4^

Nonetheless, even with all these advances, there are several effects that may adversely affect treatment delivery, such as mechanical uncertainties of the treatment machine. These include: gravity‐induced gantry and leaf bank sag, misalignment of rotational axes, as well as positioning errors of individual multi‐leaf collimator (MLC) leafs, as they affect the mechanical stability and location of the radiation central beam axis. Moreover, gravitational pull during gantry rotation affects on‐board imaging systems as well. Though flex maps are commonly used to account for the movement of the imager components, it may lead to an offset between the mechanical and the imaging isocenters.^5^, ^6^ Rigid quality control is therefore of utmost importance, and quality standards for stereotactic machines have been defined that exceed those required for conventional therapy according to TG‐142.^7^

A common method to assess mechanical imperfections affecting the isocenter is the Winston‐Lutz (WL) test. First developed in 1988 using radiochromic films,^8^ the method has become more wide‐spread after the establishment of portal imaging made the method less tedious to use.^9^, ^10^, ^11^, ^12^ Images of a ball‐bearing phantom in the radiation beam are acquired at several gantry, collimator, and table angles. Originally, the ball‐bearing phantom was iteratively moved to the center of each radiation field, with its final location corresponding to the radiation isocenter. In a later development, the ball‐bearing phantom is kept stationary and serves as a reference, whose position relative to the treatment beam is detected. This allows the check to be expanded to include other systems such as the laser isocenter and the isocenter of on‐board cone‐beam computed tomography (CT) systems.^10^, ^13^

The Winston‐Lutz test allows for the detailed acquisition of the gantry‐ and table‐dependent movement of the isocenter. These shifts are typically below 1 mm in a well‐commissioned machine and thus barely affect the delivery of standard intensity‐modulated and conformal plans. However, they can be expected to affect the target coverage for small lesions as are commonly found in stereotactic radiosurgery. While the impact of the MLC^14^, ^15^, ^16^, ^17^ and patient positioning^18^ on delivery precision has been the focus of several studies, data on the impact of isocenter shifts on target coverage are scarce. The same is true for attempts to compensate for isocenter deviations, with two notable exceptions: Rowshanfarzad et al.^19^ applied a correction for EPID and gantry sag to acquired quality assurance (QA) images, and Du et al.^20^ developed an MLC‐based strategy compensating for gantry sag through MLC movement. These studies demonstrate that isocenter corrections based on Winston‐Lutz tests are feasible and may be used to improve precision of treatment delivery.

In Section 2.A, we quantify the deviations observed at two Elekta Synergy linacs used for stereotactic treatments at our institution. Next, we validate our treatment planning system (TPS)‐based correction approach using the ArcCHECK phantom, as outlined in Section 2.B. Afterward, we demonstrate in the TPS how misalignment of the main rotational axes and a mismatch between treatment and imaging isocenters can affect planning target volume (PTV) coverage in stereotactic radiosurgery (SRS) and stereotactic radiotherapy (SRT) treatments of cerebral metastases. In Section 2.C, we investigate the impact of chosen deviations on circular cerebral lesions, both in terms of PTV coverage as well as Paddick's conformity and gradient indices.^21^, ^22^ Furthermore, we apply the shifts measured at our two linacs to patient plans treated at these machines within the last year, as described in Section 2.D.

## 2 MATERIALS AND METHODS

### 2.A Measurement of isocenter location using the Winston‐Lutz approach

Winston‐Lutz measurements were carried out on two Elekta Synergy® linacs with Agility MLC (Elekta, Stockholm, Sweden). Both machines are equipped with on‐boarding imaging, including a portal imager (Elekta iView GT, version 3.4) and kV CBCT imaging (Elekta XVI version 5.0.2). For the kV imager, two CBCTs were acquired, with filter settings F0 S10 at 120 kV and a slice thickness of 0.5 mm, with rotation directions clockwise and counterclockwise, respectively. MV images of 18 × 18 cm^2^‐sized fields at 6 MV were acquired for 20 gantry‐ and collimator angles with steps of 18 degrees, and 13 couch angles in 15° intervals ranging from 90° to 270Linda J. Wac.

**Fig. 1:**
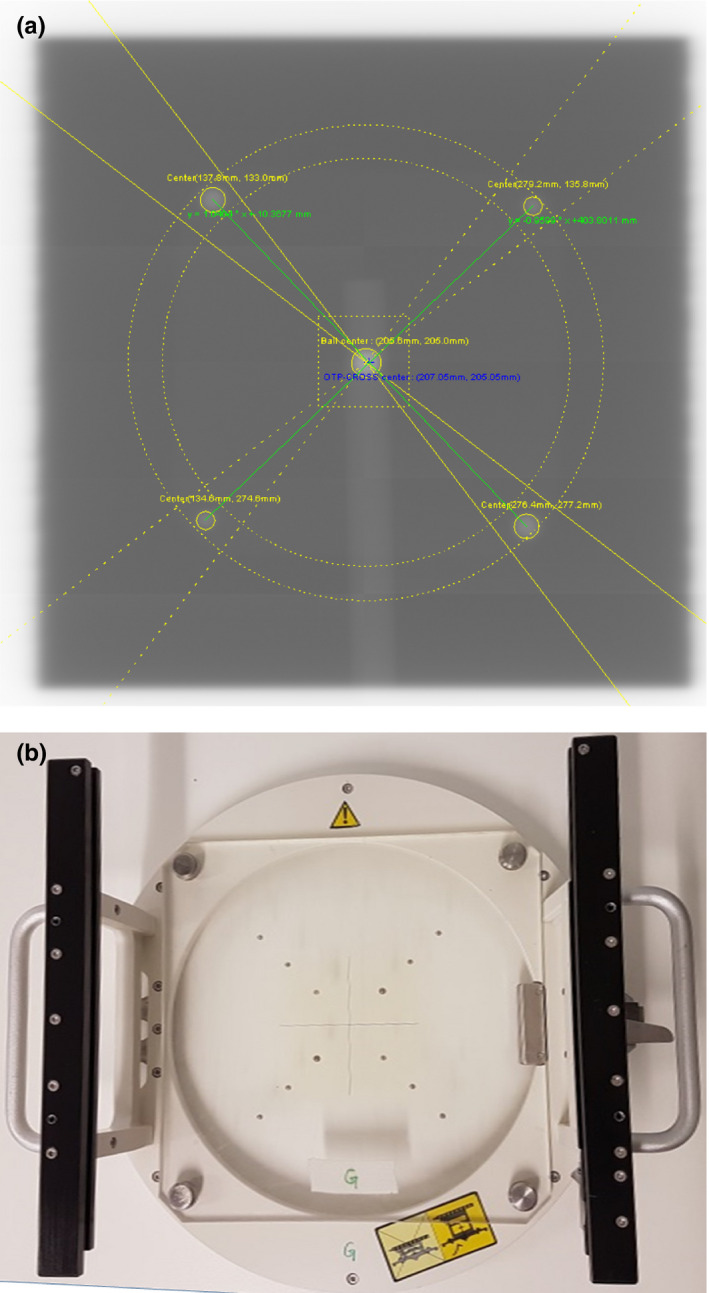
(a) Exemplary picture of the Winston‐Lutz analysis carried out in QualiFormeD: The central object is a projection of the ball‐bearing phantom, while the four peripheral balls are part of the test object fixed to the tray shown in (b).

Instead of the jaws, the intersection of four balls on an in‐house test object fixed to the shadow tray on the collimator head served as a reference for the radiation beam, while the Elekta ball‐bearing phantom aligned to the lasers served as a stationary reference (s. Fig. 1). The test object consisted of a PMMA plate with inserted steel balls of the same dimensions as the commercially available OTP‐cross test object commonly used together with the software platform QualiFormeD (La Roche Sur Yon, France).

For the analysis, we used the software module ISO‐CBCT by QualiFormeD, which is based on the method presented by Winkler et al.^10^ The software determines the relative shifts between the ball‐bearing phantom and the reference object fixed to the collimator head. Images are corrected for variations in source‐imager distance due to panel sag within the software. In‐plane shifts of the imager panel do not affect the analysis as the software only analyzes changes in the test objects relative to one another.

By performing a CBCT of the ball‐bearing phantom prior to acquiring the projections for various angles, the method allows for the detection of CBCT and MV isocenters, as well as the location of the three rotational axes (gantry, collimator, and couch). Mechanical instabilities such as axis wobble and gantry sag can be quantified as well. Directions were specified as follows: x‐axis as left‐right, y‐axis as down‐up, and z‐axis as target‐gun direction. Gantry sag is defined as a movement of the central beam axis in the z‐direction due to gantry rotation, while gantry wobble refers to movements of the central beam axis in the transversal plane during gantry rotation. The x and y coordinates of the MV isocenter in the transversal plane were set to coincide with the gantry axis. In the z‐direction, the isocenter location was set as the average of z‐coordinates of collimator axis and the table axis measured at gantry 0°.

All WL measurements were repeated twice within a 6‐month period. The shifts observed proved to be stable within that time. Static projections for WL measurements were performed for clockwise and counterclockwise rotations. As only negligible differences were observed and for the sake of brevity, only the corrections for the clockwise rotations are shown in this study. The observed differences in axis wobble amounted 0.2 mm or less, and no differences in axis location were found between the two rotational directions.

Two scripts were generated for Pinnacle^3^ (version 16.2) that can be used to apply the measured isocenter deviations on any given treatment plan. The script was able to account for the following shifts: gantry sag, the distance between CBCT and MV isocenters, and the distance of collimator and table axes to the gantry axis. Any translational and rotational deviations were incorporated into the plan by assigning a machine‐ and angle‐specific isocenter for each beam that reflected the position changes of the central beam under the given linac setup. The precision of isocenter location in Pinnacle version 16.2 is 0.1 mm. Note that, the script can only be applied to three‐dimensional (3D)‐conformal or IMRT treatment plans, but not to conformal arc or volumetric modulated arc therapy deliveries, as a distinct isocenter can only be set for each beam, but not for every control point of each beam.

### 2.B Validation using the ArcCHECK phantom

Our in‐house SRT QA check (Wegener *et al*., manuscript in progress) mimics the setup and irradiation of a spherical target of 3 cm in diameter located at the center of the ArcCHECK (Sun Nuclear Corporation, Melbourne, FL, USA), a cylindrical PMMA phantom containing a helical diode array of 1386 diodes. The test consists of a 12‐field test plan with gantry (G), collimator, and table (T) angles chosen to reflect the standard configuration used at our institution for isotropic stereotactic irradiations of intracranial targets while sparing the ArcCHECK’s electronic components: T0°G117°, T0°G79°, T0°G37°, T0°0°, T288°G37°, T324°143°, T324°G101°, T324°G63°, T36°G243°, T36°G281°, T36°G324°, and T72°G217. For each beam, 100 MU are applied. The reference dose distribution is obtained using Pinnacle^3^ (Version 16.2). While planning is carried out using an artificial CT of the ArcCHECK with a homogeneous density set to 1.15 g/cm^3^ on a dose grid of 1 × 1 × 1 mm^3^, a CT scan using a Somatom Sensation (Siemens Healthineers, Erlangen, Germany) is also carried out for image registration of subsequent cone‐beam CTs. For better image registration of the rotationally symmetric phantom, a custom‐built PMMA inset with a cross‐shaped air cavity was inserted at a fixed angle for both reference CT and later CBCT scans.

During the check, the CBCT scan of the phantom with the cross‐shaped insert is registered against the reference CT scan and positioned using iGUIDE (version 2.2) and a hexapod table (Medical Intelligence, Schwabmünchen, Germany) to account for translational and rotational setup errors.^4^ Before the measurements, the insert is replaced by the standard insert containing a 0.125 cm^3^ ionization chamber PTW Semiflex 31010 (PTW‐Freiburg, Germany). Cross‐calibration using a 10 × 10 cm^2^ field at gantry angle 0° is performed before the application of the 12‐field plan. Dose recording and subsequent comparison of the measurement to the reference dose are carried out using the software SNC patient version 6.6 (Sun Nuclear Corporation, FL, USA). For this study, three measurements were carried out on linac 1 within a 2‐month interval.

To assess the impact of errors in isocenter location, several modified reference plans were created: one for each error separately, as well as one with all errors combined. The errors accounted for were the deviation between CBCT and MV isocenters, the distance of the collimator and table axes to the gantry axis, and gantry sag. The magnitude and direction of the errors were taken from the Winston‐Lutz measurements described in Section 2.A. Gantry sag for angles not acquired during the measurements was linearly interpolated. Pass rates for Gamma 2%/2 mm were assessed for the measurements using the original as well as the modified reference plans. The minimum dose threshold to be included in the analysis was set to 10% of maximum dose. A *t*‐test for connected samples was performed to assess the differences in Gamma pass rates for the various reference plans for statistical significance.

The Gamma criteria were selected as a compromise that was sufficiently sensitive to the introduced changes without losing to much accuracy due to inadequate sampling. In Low, Dempsey,^23^ the authors recommend a pixel spacing of less than or equal to 1/3 of the distance‐to‐agreement criterion Δd, which would result in a Gamma criterion for Δd of at least 3 mm at a dose grid of 1 mm. However, the changes in isocenter position observed in this study are smaller. We therefore chose Δd = 2 mm, as notable changes are expected to occur at this scale, while not deviating from previously published recommendations.^23^, ^24^

### 2.C Planning study

On a planning CT of the head of a random patient, a hypothetical tumor location was chosen in the center of the brain located just cranially to the lateral ventricles to reduce the impact of anisotropy. Treatment plans containing 10 noncoplanar conformal static beams were generated for circular PTVs measuring 1, 2.5, and 5 cm^3^, with a dose prescription of 1 × 18 Gy to the 80% isodose. The isocenter was placed at the barycenter of the PTV, and the dose grid set to 1 × 1 × 1 mm^3^. The planning strategy aimed at an optimization of conformity index while maintaining a target volume coverage of the prescribed isodose (V_18Gy_) of at least 97%. Additionally, two more plans were generated for the 1 cm^3^ PTV: one of them exceeded the prescribed dose, resulting in a surface isodose of 18.5 Gy, while the other failed to reach the prescribed dose, with a surface isodose of 16.5 Gy. These dose levels resulted in isodose lines with a radius that was 0.5 mm wider/shorter, respectively.

A script was generated for our TPS that allowed for the introduction of the following deviations: the distance between CBCT and MV isocenters, the distance between gantry and table axes and gantry and collimator axes, as well as gantry sag. The method was the same as described in Section 2.A. For all modified plans, monitor units were set to match those assigned in the unmodified plan.

The introduced errors were chosen to cover the same order of magnitude that was commonly observed in literature.^19^ For the distance between CBCT and MV isocenters, the distance between gantry and table/collimator axis, and for gantry sag, the error range was set from 0.25 to 2.0 mm. Gantry wobble was set to be in a range of 0 to 0.3 mm, which was the largest value observed at our institution during routine QA. The results for gantry wobble are not shown in Section 3 as its impact on the results was deemed irrelevant (<0.5% change for all analyzed parameters).

The impact of the introduced isocenter shifts was quantified using the dose‐volume histogram (DVH) measures D_98_, D_95_, D_02_, target volume coverage (TVC), for example, V_18Gy_, as well as Paddick’s conformity and gradient indices, which are two widely accepted measures of plan quality in stereotactic treatment planning. Paddick’s conformity index (CI) is defined as^21^:

$$CI=\frac{PTV*PIV}{PTV_{\mathrm{PIV}}^{2}}$$

with PTV as planning target volume, PIV as the volume encompassed by the prescription isodose, and PTV_PIV_ as the PTV located within the PIV. Better conformity of the prescription isodose to the PTV will result in smaller CI, with a CI of 1 for a hypothetical ideal plan.

Paddick’s gradient index (GI) is defined as follows^22^:

$$GI=\frac{V_{X/2}}{V_{x}}$$

where V_X_ is the isodose volume of the prescription isodose and V_X/2_ is the volume receiving at least half of the prescription dose.

### 2.D Selection of clinical plans

Sixteen clinical plans of stereotactic treatments of the head that were delivered within the last 12 months at our institution were selected. PTV sizes ranged from 0.23 to 10.4 cm^3^, and dose descriptions of 5 to 30 Gy, delivered in one to six fractions.

The shifts measured for both accelerators using the Winston‐Lutz approach were applied to each treatment plan and the effect on TVC, D_98_, D_95_, and D_02_ was quantified. Differences were tested for statistical significance using a t‐test for connected samples, assuming normal distribution.

## 3 RESULTS

### 3.A Winston‐Lutz measurements

The Winston‐Lutz test results are listed in Table 1. For the location of the CBCT isocenter, both machines are well within tolerances, with deviations of <0.5 mm in each direction. In case of linac 2, this is also true for the table and collimator axes, while for linac 1, we observed a distance of 1.2 mm between table and gantry axes, and a distance of 0.6 mm between collimator and gantry axes.

**Table 1:** Offsets in mm for cone‐beam computed tomography isocenter, table, and collimator rotation axis from the MV isocenter. Origin is set to MV isocenter, defined as the point on the gantry axis closest to table and collimator axes.

	Direction	Linac 1 (mm)	Linac 2 (mm)
CBCT	Left(−)/right(+)	−0.1	−0.3
	Down(−)/up(+)	0.3	0.2
	Target(−)/gun(+)	−0.2	0.0
Table	Left(−)/right(+)	1.2	0.3
	Target(−)/gun(+)	−0.2	0.2
Collimator	Left(−)/right(+)	0.6	0.0
	Target(−)/gun(+)	0.2	0.2
Gantry sag (amplitude)	Target(−)/gun(+)	1.3	1.1

### 3.B Validation using the ArcCHECK phantom

**Fig. 2:**
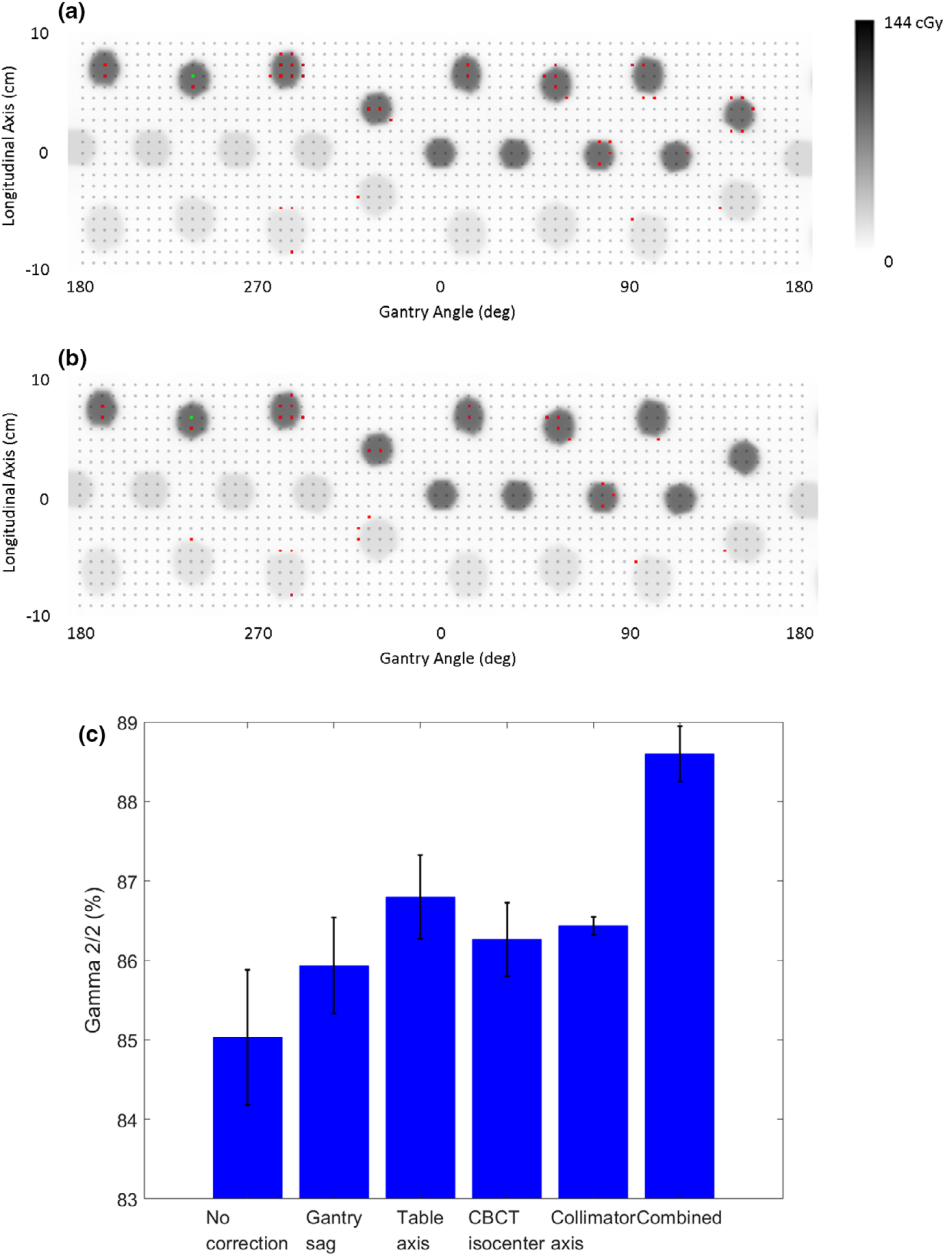
ArcCHECK diode analysis for one measurement using a reference with no corrections (a), with corrections (b), and (c) mean pass rates for Gamma 2%/2 mm for each correction individually, as well as all corrections combined. Images show the dose distribution on the ArcCHECK phantom mantle. Gray points indicate diode locations, with failing diodes highlighted in red. The green point indicates the dose maximum. Error bars indicate standard deviations observed for three measurements.

Performing the SRT QA check results in a mean absolute pass rate of (85.0 ± 0.85)% at Gamma 2%/2 mm averaged over three measurements. The distribution of passing and failing diodes along the ArcCHECK mantle at Gamma 2%/2 mm for the unmodified reference of a representative measurement is shown in Fig 2(a). Figure 2(b) shows the result for the same measurement, but with a modified dose distribution accounting for CBCT isocenter offset, gantry sag, and misalignments of table and collimator axes as expected from the WL test results. A closer look reveals that beams applied at gantry angles between 90° and 180° showed the most significant improvements, particularly the beam T324°143°, which includes both large gantry and table rotations, as shown on the far right in Figs. 2(a) and 2(b). Figure 2(c) shows the improvements of Gamma 2%/2 mm pass rates when isocenter deviations are accounted for in the reference, both for each correction individually as well as all corrections combined. The mean increase in Gamma pass rate for a single correction ranges from 0.9% (gantry sag) to 2.8% (coincidence of gantry and table axes). With all corrections applied, mean pass rates rise to (88.6 ± 0.34)% (*P* = 0.003).

### 3.C Planning study

**Fig. 3:**
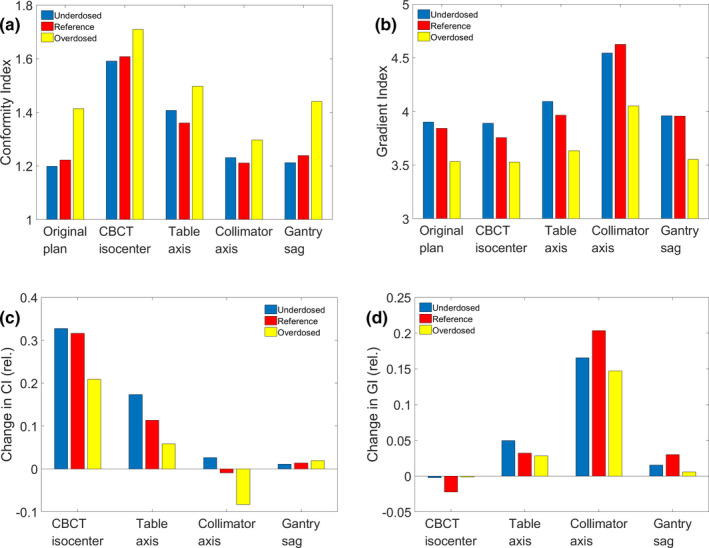
Changes in conformity (a) and gradient index (b), for reference, underdosed, and overdosed plans for shifts of 1.5 mm in cone‐beam computed tomography isocenter, gantry sag, and collimator and table axes location. Below, the relative differences in conformity (c) and gradient index (d) due to isocenter shifts normalized to the respective original plans are shown.

Figure 3 shows the changes in CI and GI for the 18 Gy isodose on a 1 cm^3^‐sized spherical target caused by a 1.5‐mm error in the location of CBCT isocenter, table and collimator axes, as well as a 1.5‐mm amplitude of gantry sag. The results are shown for the optimal reference plan, as well as the overdosed and underdosed plans.

Conformity and GI for the 18 Gy isodose were evaluated. The overdosed reference plan results in a worse CI [Fig. 3(a)] but better GI [Fig. 3(b)] than the reference plan. The opposite was the case for the underdosed plan (better CI, worse GI).

Figure 3(a) and 3(c) show that CI is most vulnerable to large distances between CBCT and MV isocenters and less affected by errors in the position of the table axis. It remains mostly unaffected by errors in collimator axis position and gantry sag. Interestingly, while the underdosed and reference plans start out with better values than the overdosed plan [Fig. 3(a)], shifts in isocenter position lead to more severe relative deterioration than in overdosed plans [+32,7% and +31,6% vs 20,9% for a 1.5 mm shift in CBCT isocenter location, Fig. 3(c)].

Gradient index worsened when the 1.5‐mm distance was applied between the collimator and gantry axes, while being robust to shifts in CBCT isocenter position and gantry sag. The overdosed plans start out with better GI values. They also appear to be more robust to changes in isocenter location. A 1.5‐ mm distance between collimator and gantry axes results in a 14.7% and 20.4% increase in GI for the overdosed and the reference plans, respectively [Fig. 3(d)].

**Fig. 4:**
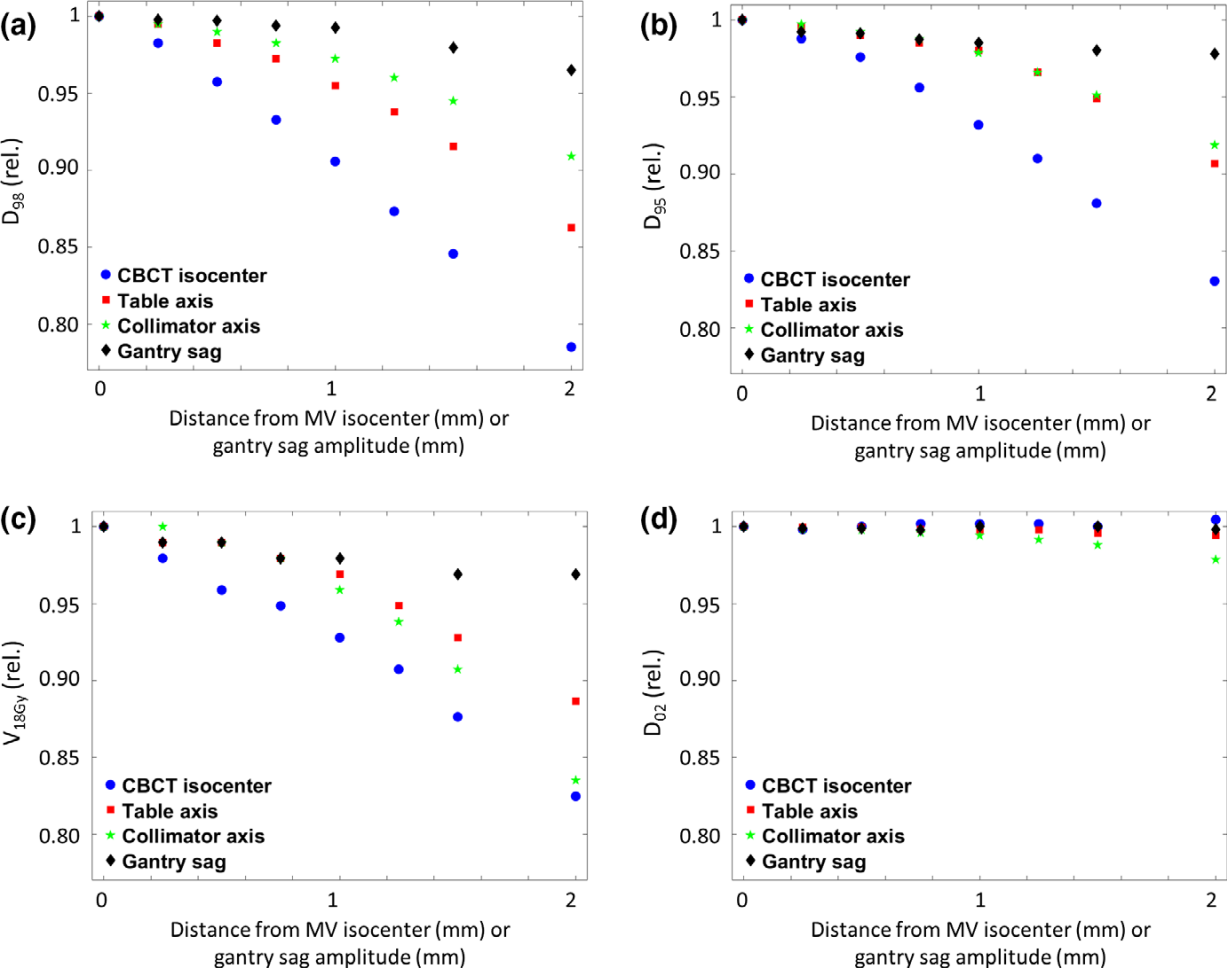
Changes in D_98_ (a), D_95_ (b), V_18Gy_ (c), and D_02_ (d) caused by different isocenter deviations or gantry sag amplitudes in a 1 cm^3^ spherical target volume, normalized to the unmodified reference plan.

The impact on the DVH characteristics for a 1 cm^3^‐sized spherical PTV shows similar trends for D_98_ [Fig. 4(a)], D_95_ [Fig. 4(b)], and V_18Gy_ [Fig. 4(c)]. For all of them, an error in CBCT isocenter location has the strongest impact (−11.5% for D_98_ at 2‐mm distance), while even large amplitudes of gantry sag lead to negligible changes in these measures (−3.5% at 2‐mm amplitude). V_18Gy_ dropped by 16.5% for a 2 mm shift of CBCT isocenter, while even a shift of 1 mm, which is considered within tolerance according to TG‐142, lead to a drop of 9.4% in D_98_ and 7.2% in V_18Gy_. Increasing the distance between table and collimator axes to the gantry axis lead to less severe but relevant decrease in D_98_ (−10.9% and −9.1% at 2‐mm distance, respectively). For an offset of 1 mm of collimator and table axes to the gantry axis, changes in D_98_ were less concerning (−4.5% and −2.75% for table‐gantry and collimator‐gantry axes distance, respectively).

For D_02_, no major changes were observed for any of the introduced errors. The only relevant change observed was a slight drop of −2.1% for gantry‐collimator axis distance of 2 mm [Fig. 4(d)].

**Fig. 5:**
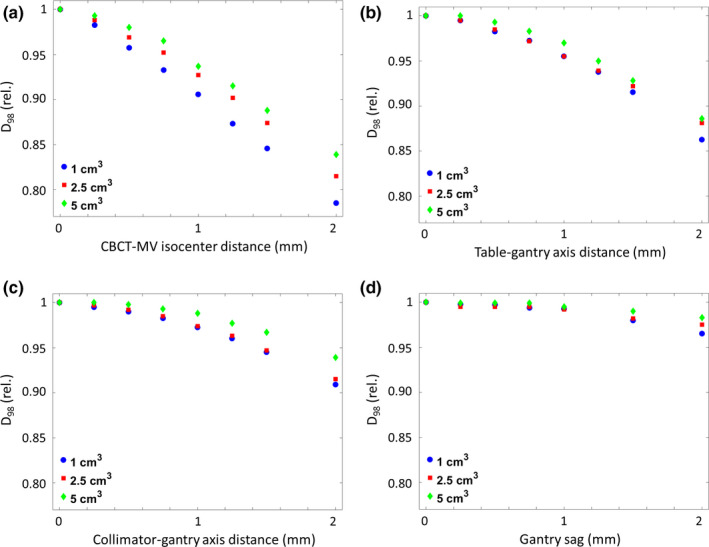
Changes of D_98_ for spherical volumes of varying size for errors in cone‐beam computed tomography (a), table axis (b), and collimator axis (c) location, as well as gantry sag (d).

Larger spherical volumes are less severely affected by isocenter shifts with increasing size, though trends observed for the 1 cm^3^ volume remain. Figure 5 shows the changes in D_98_ for PTV volumes measuring 1, 2.5, and 5 cm^3^ after shifts in CBCT isocenter [Fig. 5(a)], table axis [Fig. 5(b)], and collimator axis [Fig. 5(c)]. For a 1‐mm distance between CBCT and MV isocenters, D_98_ dropped by 8.3% and 6.3% for the 2.5 and 5 cm^3^ PTVs, respectively. Similar trends were observed for deviations of 1 mm in table axis (−4.5% and −3.0%) and collimator axis (−2.6% and −1.2%) distance. A gantry sag of 1 mm resulted in barely discernable differences in D_98_ (−0.8% and −0.5%) for larger volumes of 2.5 and 5 cm^3^. At a gantry sag of 2 mm, however, differences in the reference plan increased to −2.5% and 1.7%, respectively.

**Fig. 6:**
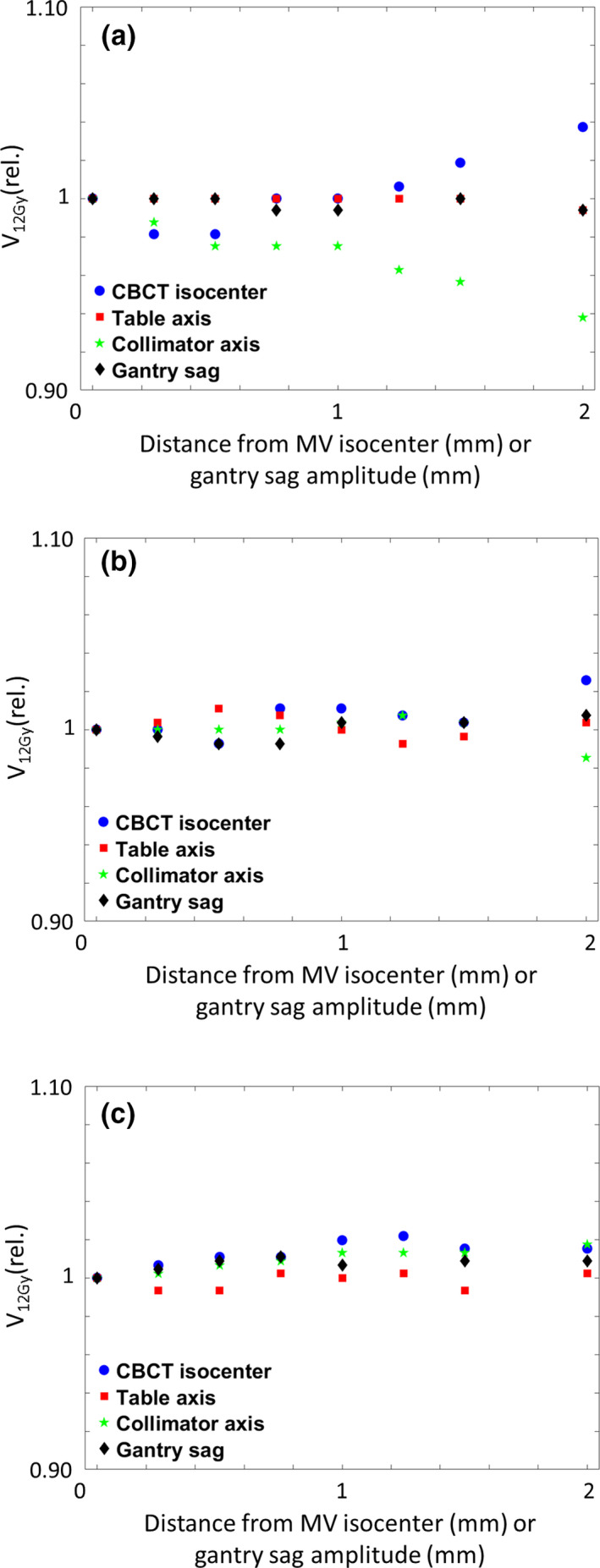
Changes in V_12Gy_ to healthy brain tissue caused by different isocenter deviations for spherical volumes of (a) 1 cm^3^, (b) 2.5 cm^3^, and (c) 5 cm^3^, normalized to their respective unmodified reference plans.

Figure 6 shows changes in V_12Gy_ to healthy brain tissue for PTV volumes of 1, 2.5, and 5 cm^3^, normalized to their respective reference plans. For a PTV of 1 cm^3^, changes in CBCT isocenter position of 2 mm resulted in a relevant increase (+3.7%) of V_12Gy_ to normal brain tissue [Fig. 6(d)], while changes in collimator axis position lead to a decrease of 6.2%. For larger volumes, changes in V_12Gy_ showed no clear trends [Figs. 6(b) and 6(c)], with changes of no more than 2.5% for any introduced shift.

### 3.D Impact of isocenter shifts on clinical plans

**Fig. 7:**
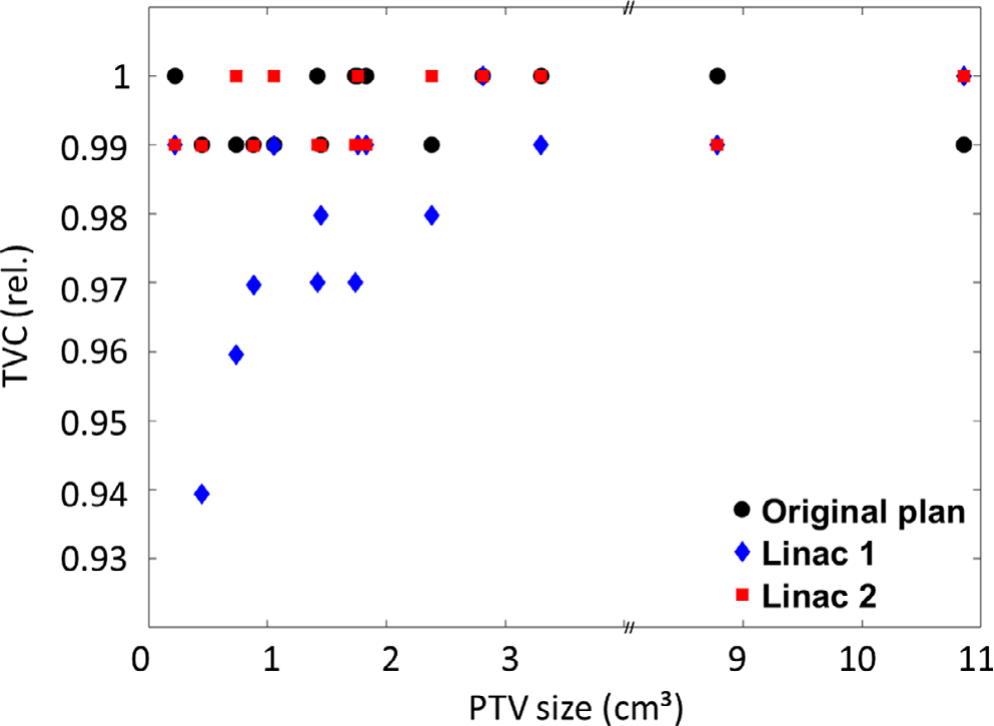
PTV coverages (TVC) of the original plans and after isocenter misalignments as measured for linac 1 and linac 2.

Results for TVC of 16 patient plans before and after applying the shifts listed in Table 1 are shown in Fig. 7. The shifts observed on linac 2 (red) do not cause a relevant decrease in target coverage even for the smallest PTVs. However, linac 1 (blue) performed significantly worse than linac 2 (*P* = 0.003) due to the larger distance between gantry and table axes, which leads to a larger table run out. In targets smaller than 1 cm^3^, a drop in TVC coverage of up to 6% is observed. Similar observations were made for D_98_ (not shown, *P* = 0.001). Plans that had a TVC of 100% of the prescription dose were as susceptible to errors in isocenter location as plans with a lower initial TVC (*P* = 0.62).

## 4 DISCUSSION

The isocenter shifts investigated in this study have different effects on dose distribution. A displacement of CBCT isocenter will result in a shift of the dose distribution, but little to no impact on dose gradients and maximum dose. Gantry sag and a shift of the collimator axis, however, will result in a more or less isotropic blurring of the dose distribution. A misalignment of the table axis is ranged somewhere between these effects, as table rotations on Elekta linacs commonly cover only a range of −90° to 90°, resulting in anisotropic blurring.

The Winston‐Lutz approach used in this study utilizes a reference object fixed to the collimator head rather than the MLCs and jaws, excluding additional shifts in the central beam axis caused by sagging of the jaws and MLC. Therefore, the test is suitable to detect deviations of the rotational axes, but not errors due to beam steering or jaw positioning. However, jaw and leaf bank sagging also depends on collimator angle, and during stereotactic radiotherapy, different collimator angles can be used. This approach reflects the procedure used during linac commissioning, where the mechanical isocenter is found, and then beam steering, MLC, and jaws are calibrated accordingly.

Our results (Fig. 3) confirm a finding already described by Ohtakara et al.^25^ They found that for isodose surface‐based plans, CI increased when higher percentage isodoses were chosen, while GI decreased, effectively leading to plans with better gradients, but worse conformity. Moreover, the blurring effect of collimator axis misalignment resulted in changes similar to those observed for underdosed plans (increasing GI with slightly reduced CI), while for CBCT isocenter shifts, GI remained the same with worse CI. Misalignments of the table axis showed a combination of the two effects, with visible but less severe deterioration of GI and CI.

Our results indicate that intervention thresholds of 1 mm (DIN 6847‐5, TG‐142), which are acceptable for conventional therapy, might be too large for machines used for stereotactic irradiation of very small target volumes, particularly for the distance between MV and CBCT isocenters. If possible, any deviations, both for axis misalignment and imaging isocenter, should be kept below 0.5 mm, as our results indicate that these deviations do not have a relevant impact on treatment quality even for the smallest lesions. This conclusion is supported by Fig. 6, which shows that patient plans treated on a linac within these tolerances did not result in deterioration of PTV coverage.

The gantry sag of 1.3 mm measured at our linacs is consistent with published results on Elekta machines.^19^ This is larger than for Varian machines, which typically present with a gantry sag of about 0.8 mm.^10^, ^13^ However, according to our results, gantry sag has less of an impact on treatment accuracy, even with an amplitude of 1.5 mm.

Of course, it must be pointed out that we did not cover a large number of other potential uncertainties, many of which may have a more severe impact on treatment quality. These include MLC‐based uncertainties such as MLC sag and leaf positioning errors, as well as errors in target delineation and patient setup.

Our method offers the potential to predict the impact of isocentric shifts on a given clinical plan, which would be advised for plans with small target volumes treated at machines with known isocenter offsets exceeding 0.5 mm. The feasibility of this was demonstrated by applying a stereotactic plan to the ArcCHECK phantom, where Gamma pass rates increased when the reference dose distribution was altered to account for known isocenter deviations.

As gantry sag, the location of the axes, and the CBCT isocenter are quite constant over time, isocentric shifts can be mitigated in the planning process by adjusting the isocenter location of each beam in the same fashion our scripts do with no changes to linac hardware. However, this would require more elaborate clinical validation.

## 5 CONCLUSION

While usually irrelevant for conventional therapy, isocentric shifts may have a clinically relevant impact on treatment quality of irradiations of stereotactic PTVs smaller than approximately 2 cm^3^. The method we presented allows for the quantification and correction of errors in isocenter location caused by an offset between CBCT and MV isocenters, misalignment of linac rotation axes, and gravitationally induced gantry sag.

## CONFLICT OF INTEREST

This publication was funded by the German Research Foundation (DFG) and the University of Wuerzburg in the funding program Open Access Publishing.
